# Beneficial In Vitro Effects of a Low *Myo*-Inositol Dose in the Regulation of Vascular Resistance and Protein Peroxidation under Inflammatory Conditions

**DOI:** 10.3390/nu14051118

**Published:** 2022-03-07

**Authors:** Agata Rolnik, Beata Olas, Joanna Szablińska-Piernik, Lesław Bernard Lahuta, Andrzej Rynkiewicz, Piotr Cygański, Katarzyna Socha, Leszek Gromadziński, Michael Thoene, Michał Majewski

**Affiliations:** 1 Department of General Biochemistry, Faculty of Biology and Environmental Protection, University of Łódź, 90-236 Łódź, Poland; agata.rolnik@edu.uni.lodz.pl (A.R.); beata.olas@biol.uni.lodz.pl (B.O.); 2 Department of Plant Physiology, Genetics and Biotechnology, University of Warmia and Mazury in Olsztyn, 10-719 Olsztyn, Poland; joanna.szablinska@uwm.edu.pl (J.S.-P.); lahuta@uwm.edu.pl (L.B.L.); 3 Department of Cardiology and Internal Medicine, Faculty of Medicine, University of Warmia and Mazury in Olsztyn, 10-082 Olsztyn, Poland; andrzej.rynkiewicz@uwm.edu.pl (A.R.); piotr.cyganski@uwm.edu.pl (P.C.); leszek.gromadzinski@uwm.edu.pl (L.G.); 4 Department of Bromatology, Medical University of Białystok, 15-222 Białystok, Poland; katarzyna.socha@umb.edu.pl; 5 Department of Medical Biology, Faculty of Health Sciences, University of Warmia and Mazury in Olsztyn, 10-561 Olsztyn, Poland; michael.thoene@uwm.edu.pl; 6 Department of Pharmacology and Toxicology, Faculty of Medicine, University of Warmia and Mazury in Olsztyn, 10-082 Olsztyn, Poland

**Keywords:** acetylcholine, hydrogen peroxide, *myo*-inositol, nitric oxide, pinacidil, reactive oxygen species, thromboxane A_2_, U-46619

## Abstract

Oxidative stress induces functional changes in arteries. Therefore, the effect of *myo*-inositol, a possible anti-inflammatory/antioxidant agent was studied on human plasma and rat thoracic arteries. Aortic rings from male Wistar rats (3 months of age) were incubated with *myo*-inositol (1, 10 and 100 μM, 120 min) and analyzed using the gas chromatography (GC) method. In another experiment, aortic rings were protected first with *myo*-inositol (1 µM, 60 min) and then subjected to a thromboxane receptor agonist (U-46619, 0.1 nM, 60 min). Therefore, these four groups under the following conditions were studied: (i) the control in the vehicle; (ii) *myo*-inositol; (iii) the vehicle plus U-46619; (iv) *myo*-inositol plus U-46619. The hemostatic parameters of human plasma and an H_2_O_2_/Fe^2+^ challenge for lipid and protein peroxidation were also performed. *Myo*-inositol was not absorbed into the pre-incubated aortic rings as measured by the GC method (0.040 µg/mg, *p* ≥ 0.8688). The effect of *myo*-inositol was more significant in the impaired arteries due to U-46619 incubation, which resulted in an improved response to acetylcholine (% Emax: 58.47 vs. 86.69), sodium nitroprusside (logEC_50_: −7.478 vs. −8.076), CORM-2 (% Emax: 44.08 vs. 83.29), pinacidil (logEC_50_: −6.489 vs. −6.988) and noradrenaline (logEC_50_: −7.264 vs. −6.525). This was most likely a possible response to increased nitric oxide release (×2.6-fold, *p* < 0001), and decreased hydrogen peroxide production (×0.7-fold, *p* = 0.0012). KCl-induced membrane depolarization was not modified (*p* ≥ 0.4768). Both the plasma protein carbonylation (×0.7-fold, *p* = 0.0006), and the level of thiol groups (×3.2-fold, *p* = 0.0462) were also improved, which was not significant for TBARS (×0.8-fold, *p* = 0.0872). The hemostatic parameters were also not modified (*p* ≥ 0.8171). A protective effect of *myo*-inositol was demonstrated against prooxidant damage to human plasma and rat thoracic arteries, suggesting a strong role of this nutraceutical agent on vasculature which may be of benefit against harmful environmental effects.

## 1. Introduction

*Myo*-inositol is a cyclic six-carbon polyalcohol found in cell membranes, is synthesized from glucose and is classified as a cyclitol. Furthermore, there are other similar molecules of the same class that have also shown physiological benefits. The other cyclitols that have been studied lately for their physiological effects include D-*chiro*-inositol and its methyl derivative—D-pinitol [1,2]. However, less attention has been paid to *myo*-inositol. *Myo*-inositol exists in the cell membrane in a phosphorylated form where it acts as a messenger molecule regulating the actions of many hormones including insulin, follicle-stimulating hormone, and thyrotropin [2]. Furthermore, *myo*-inositol may also be found in its free form or a derivative form, where it can act upon various processes including stress responses, metabolic homeostasis, the permeability of ion channels and even the regulation of mRNA. *Myo*-inositol has also been shown to have anti-inflammatory/antioxidant capabilities and is a possible vasodilator [3,4] with a good therapeutic option in the reduction of metabolically induced inflammation [5]. These properties may provide protection to the vascular system, which is the focus of this study. 

Some of the metabolic functions that *myo*-inositol seems to effect have been well documented, but the mechanisms are lacking. Much of the current research has focused upon *myo*-inositol as a dietary supplement to help control various disorders, such as type 2 diabetes, gestational diabetes and has shown promise in helping to alleviate polycystic ovary syndrome (PCOS) [2,6,7,8,9,10]. However, there is still much debate concerning the effectiveness of *myo*-inositol as a dietary supplement for these disorders. *Myo*-inositol has also been shown to protect the kidney from various toxins, such as cadmium [11], and it is believed that the antioxidant ability of *myo*-inositol was the protective mechanism behind this finding. There have been other studies as well that have shown *myo*-inositol providing a protective effect against inflammation induced by endothelial dysfunction (ED) in diabetes [4]. *Myo*-inositol has been shown to provide a scavenging effect toward reactive oxygen species (ROS) in vitro [4] and has been shown to help preserve nitric oxide (NO) signaling under diabetic conditions [12].

Cardiovascular diseases are the leading cause of death in both the developed and the developing world. According to the WHO and others, approximately 17.9 million deaths occur each year due to cardiovascular disease with one-third of these deaths occurring in people younger than seventy years of age [13]. Other studies have shown that signaling molecules, such as docosahexaenoic acid (DHA), eicosapentaenoic acid (EPA) [14] and linoleic acid (LA) [15], improve vascular response which may even offset the actions of vasoconstrictors such as U-46619, which is a stable synthetic prostaglandin (PG) H_2_ analog and thromboxane (TxA_2_) receptor agonist [16]. Therefore, this study sought to investigate if *myo*-inositol may have a similar effect upon the vascular system and ROS degradation since TxA_2_ has been correlated with the production of ROS and NO, and has been associated with vascular remodeling when maintained for a prolonged period of time.

There have also been a few studies that have suggested *myo*-inositol provides a protective effect to the cardiovascular system, but no studies have directly tested this hypothesis. One study showed that *myo*-inositol improved lipid profiles alongside its anti-diabetic effects [2], while other inositols such as D-*chiro*-inositol (DCI) and 3-*O*-methyl DCI (pinitol) helped preserve NO signaling under adverse in vitro conditions [12]. These findings point toward a potential protective effect that may be provided by *myo*-inositol. In other studies, D-pinitol was again shown to preserve NO signaling and potentiated a vasodilator effect via endothelial NO synthase (eNOS) activation in mouse mesenteric artery [1]. Another indicator that *myo*-inositol may play a role in cardiovascular protection is that adrenaline reduces the amount of inositol phosphate present in arteries and veins by hydrolysis, thus causing vasoconstriction [17]. Since adrenaline is a well-known vasoconstrictor, the hydrolysis of *myo*-inositol indicates that adrenaline is actively removing *myo*-inositol to counter the effects of vasodilation. Moreover, D-pinitol reduces the systolic blood pressure in normotensive mice [1], whereas DCI increases the action of insulin in women with PCOS, and decreases serum androgen concentrations, blood pressure, and plasma triglyceride concentrations [18]. Fruzzetti et al. [19] have shown that *myo*-inositol (4 g daily) is as effective as metformin in improving the clinical and metabolic profile of PCOS. With the above-mentioned studies in mind, it was decided to directly study the protective effects of *myo*-inositol on the thoracic arteries of young Wistar rats, as a model of the human vascular system, under normal conditions and also under adverse conditions induced by U-46619, which resemble vascular disorders observed in metabolic diseases [16]. Moreover, the influence of *myo*-inositol on lipid and protein peroxidation and hemostasis was studied in vitro.

## 2. Materials and Methods

### 2.1. Drugs and Chemicals

The following drugs were used: acetylcholine chloride (ACh), sodium nitroprusside (SNP), noradrenaline hydrochloride (NA) (Sigma-Aldrich, St. Louise, MO, USA); potassium chloride (KCl) (Chempur, Piekary Slaskie, Poland); pinacidil and U-46619 (Cayman Chemical, Ann Arbor, MI, USA). The stock solutions (10 mM) of drugs were prepared in distilled water, except for Noradrenaline (NA) which was dissolved in a NaCl (0.9%) + ascorbic acid (0.01% *w*/*v*) solution; pinacidil was dissolved in DMSO, and U-46619 in ethanol. These solutions were kept at −20 °C, and appropriate dilutions were made in KHS (in mM: NaCl 115; CaCl_2_ 2.5; KCl 4.6; KH_2_PO_4_ 1.2; MgSO_4_ 1.2; NaHCO_3_ 25; glucose 11.1) on the day of the experiment. At a concentration of 0.01%, the solvents did not alter the reactivity of isolated aortic rings.

*Myo*-inositol, trichloroacetic acid (TCA), thiobarbituric acid (TBA), sodium dodecyl sulfate (SDS), ethylenediaminetetraacetic acid (EDTA), 5,5′-dithiobis-(2-nitrobenzoic acid) (DTNB), 1-(trimethylsilyl) imidazole, pyridine and hydrogen peroxide (H_2_O_2_) were purchased from Sigma-Aldrich (St. Louis, MO, USA); while ethanol was purchased from Stanlab (Lublin, Poland). Other reagents were of analytical grade and were provided by commercial suppliers, including POCh (Gliwice, Poland), and Chempur (Piekary Slaskie, Poland). 

### 2.2. Animal Studies

Young male Wistar rats (3 months of age) were injected with intraperitoneal ketamine (100 mg/kg BW) and xylazine (10 mg/kg BW) and were then subsequently decapitated. The thoracic aorta was carefully dissected, cut into 4–5-mm rings and placed in ice-cold KHS. For gas chromatography with flame-ionization detection (GC-FID), aortic rings were incubated either with *myo*-inositol solution (1, 10 or 100 μM) or the vehicle (KHS) for 120 min. For the other experiments, aortic rings were divided into the two groups according to the presence or absence of *myo*-inositol (1 µM, 60 min). Next, aortic rings were subjected to U-46619 (0.1 nM, 60 min), which was added into the incubation chambers. Thus, these four groups were studied: (i) the control in KHS (120 min); (ii) *myo*-inositol (1 µM, 120 min); (iii) the vehicle (KHS, 120 min) plus U-46619 (60 min together with KHS); and (iv) *myo*-inositol (120 min) plus U-46619 (60 min together with *myo*-inositol). 

#### 2.2.1. Gas Chromatography with Flame-Ionization Detection of *Myo*-Inositol

After pre-incubation with either the *myo*-inositol solutions (1, 10 and 100 μM, 120 min) or the vehicle (KHS) and subsequent washout in KHS, aortic rings were placed into 200 mL of 50% ethanol solution and heated at 90 °C for 30 min with continuous shaking. After centrifugation (20,000× *g* at 4 °C, 20 min), all the supernatant with 10 μL of xylitol as an external standard (at a concentration of 10 mg/mL) was concentrated into 2 mL chromatographic vials (containing 350 μL glass inserts) in a vacuum rotary evaporator to dryness. Dry samples were derivatized in a mixture of 1-(trimethylsilyl) imidazole: pyridine (1:1, *v:v*) at 80 °C for 45 min and TMS-derivatives were analyzed with the high-resolution gas chromatography (GC) method. 

The presence of *myo*-inositol in aortic rings was monitored using a GC with a flame-ionization detector (GC-FID, GC2010 Plus, Shimadzu, Kyoto, Japan) equipped with a Zebron ZB-1 capillary column (15 m length, 0.25 mm diameter, 0.1 μm film thickness, Phenomenex, Torrance, CA, USA). The injector temperature was 325 °C and the samples were loaded onto the column using the split method (10:1). Helium was used as a carrier gas (at a flow rate of 1.18 mL/min). The column oven was heated at a programmed temperature: initial temperature 150 °C increased by 20 °C/min to 200 °C and then by 30 °C/min to 300 °C. The total analysis time was 5.83 min. The temperature of the detector was 350 °C. Data were collected and analyzed using LabSolutions software (Shimadzu, Kyoto, Japan).

#### 2.2.2. Vascular Reactivity Studies

Briefly, aortic rings from rat thoracic aorta (12.550 ± 2.065 mg) were mounted in stagnant 5 mL organ baths (Graz Tissue Bath System, Barcelona, Spain) filled with KHS being continuously aerated with carbogen and subjected to a preload tension of 1 cN (TAM-A Hugo Sachs Elektronik, March, Germany). The aortic rings were checked with KCl (75 mM KCl) and ACh (10 µM) for their functional integrity. Then, the cumulative doses of ACh (0.1 nM–10 µM), SNP (0.01 nM–10 µM), CORM-2 (1–100 µM), and pinacidil (0.1–100 µM) were added into the incubation chambers with pre-contracted (with 0.1 µM NA) aortic rings. The vasoconstrictor response was also analyzed by a single dose of KCl (75 mM), and by cumulative concentrations of NA added into the bath chambers (0.1 nM–10 μM). Only one cumulative concentration-response curve (CCRC) was performed on each aortic ring.

#### 2.2.3. Nitric Oxide Release

Briefly, during the last incubation period with either *myo*-inositol, KHS, or U-46619 the incubation fluid (0.2 mL) was collected to measure the basal NO release. The absorbance was measured at 540 nm with a nitrite colorimetric assay kit (Cayman Chemicals). The results are expressed as relative values compared to the control conditions. 

#### 2.2.4. Detection of Hydrogen Peroxide 

The production of hydrogen peroxide in the isolated aortic rings was measured with a fluorescence assay kit (Cayman Chemicals) at 530/590 nm excitation/emission wavelengths after a 60 min incubation at 37 °C in the dark, according to the instructions. Some of the rings were subjected to catalase, an H_2_O_2_ scavenger, to ensure the specificity of the method. The results are expressed as nanomoles per microgram of protein based on the standard curve with fresh H_2_O_2_ in the reaction buffer. 

### 2.3. In Vitro Studies on the Human Plasma

Human blood was obtained from nine regular donors (non-smoking men and women) of the Medical Center in Lodz, Poland. Blood was collected into tubes with CPDA solution (citrate/phosphate/dextrose/adenine; 8.5:1; *v*/*v*; blood/CPDA). Donors had not taken any medication or addictive substances (including tobacco, alcohol, and antioxidant supplementation) for a week before donation. The plasma was isolated by differential centrifuging.

#### 2.3.1. Markers of Oxidative Stress

Human plasma was pre-incubated (5 min, at 37 °C) with *myo*-inositol and treated with 4.7 mM H_2_O_2_/3.8 mM, FeSO_4_/2.5 mM EDTA (25 min, at 37 °C).

The level of lipid peroxidation was determined with the concentration of thiobarbituric acid reactive substances (TBARS), and the TBARS concentration was calculated based on absorbances measured at λ = 575 nm using the SPECTROstar Nano Microplate Reader (BMG LABTECH, Ortenberg, Germany) and expressed as nmol/mL of plasma.

The level of protein carbonylation in plasma was determined according to Levine et al. [20], while the carbonyl group concentration was calculated using a molar extinction coefficient (ε = 22,000/M cm) and was expressed as nmol/mg of plasma protein.

The level of thiol groups in protein plasma was determined spectrophotometrically using a SPECTROstar Nano Microplate Reader (BMG LABTECH). The thiol group concentration was calculated using a molar extinction coefficient (ε = 13,600/M cm) and was expressed as nmol/mg of plasma protein. The method was described by Ando and Steiner [21].

#### 2.3.2. Hemostasis Parameters 

The plasma was incubated for 30 min at 37 °C with *myo*-inositol. The prothrombin time (PT), the thrombin time (TT), and the activated partial thromboplastin time (APTT) were studied with the coagulometric method described by Malinowska et al. [22] using an Optic Coagulation Analyser, model K-3002 (Kselmed, Grudziadz, Poland). 

### 2.4. Data Analysis and Statistics

Vascular contraction induced by high KCl (75 mM) was measured in mg of developed tension, and as a % of the KCl-induced response for NA. Vascular relaxation to ACh, SNP, CORM-2, and pinacidil was measured as the % of contractile response to the vasoconstrictor NA (0.1 µM). For the CCRCs, the maximal response (Emax, %) and the potency (logEC_50_) were calculated based on a nonlinear regression model. Both the Gaussian distribution and the equality of variance were checked first. The statistical analysis was performed by either a parametric (unpaired t test, one-way ANOVA) or nonparametric (Mann Whitney) test. Results are expressed as means ± SD (and means ± SEM for CCRCs). This research was randomized and stayed blinded for laboratory analyses. The level of significance was when *p* < 0.05. 

## 3. Results

### 3.1. Gas Chromatography with Flame-Ionization Detection of Myo-Inositol in Rat Arteries

**Figure 1:**
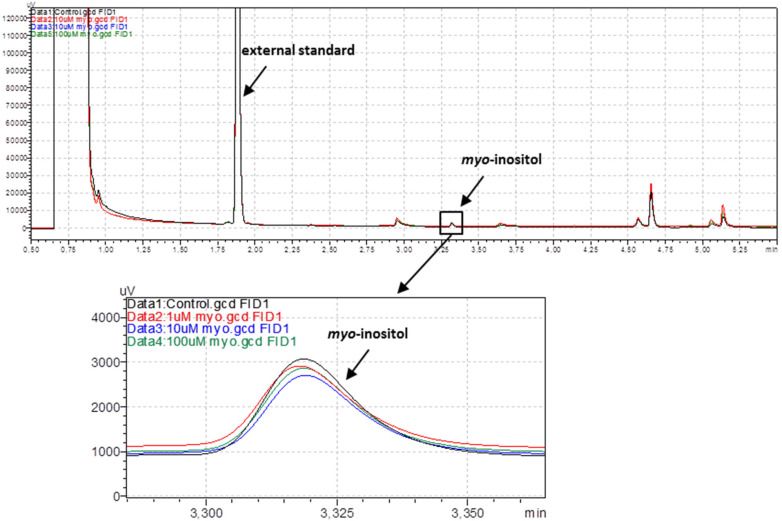
Presence of *myo*-inositol (0.040 ± 0.002 µg/mg) in aortic rings (12.55 ± 2.065 mg) analyzed with a gas chromatograph with flame-ionization detector. Control—black line; samples after pre-incubation in *myo*-inositol solutions (120 min): 1 μM—red line, 10 μM—blue line, 100 μM—green line.

*Myo*-inositol concentrations were not significantly different between the pre-incubated groups and the controls measured with the high-resolution gas chromatography method (0.040 ± 0.002 µg/mg, *p* ≥ 0.8688), see Figure 1.

### 3.2. Vascular Reactivity Studies

**Figure 2:**
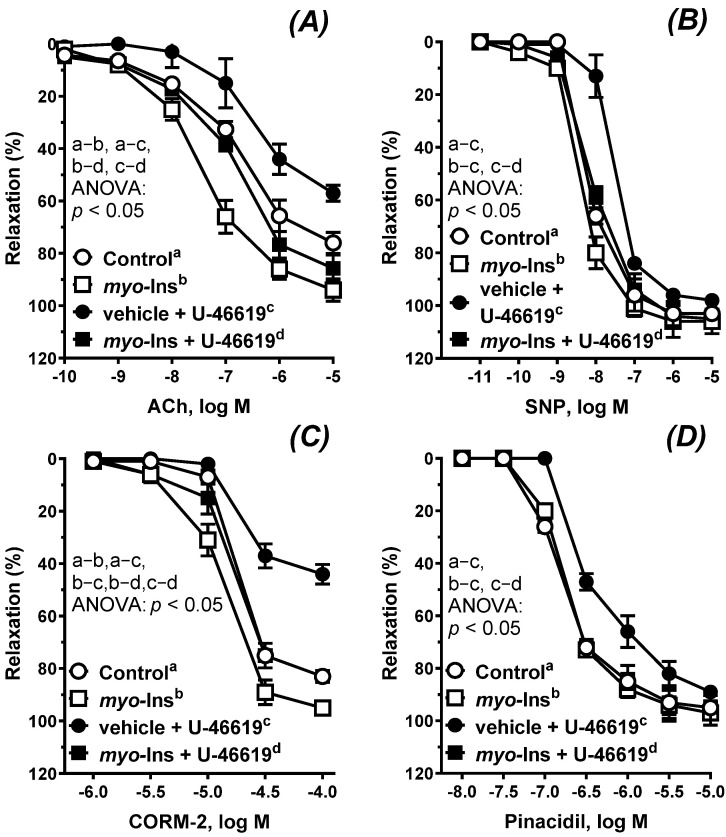
The cumulative concentration-response curves to acetylcholine (**A**), sodium nitroprusside (**B**), carbon monoxide releasing molecule (**C**), and pinacidil (**D**) in the isolated thoracic arteries of the control (a), *myo*-inositol only (b), vehicle plus U-46619 (c), *myo*-inositol plus U-46619 (d). Aortic rings were pre-incubated with either *myo*-inositol (1 µM, 60 min) for groups b and d or vehicle (KHS)—groups a and c. Next, aortic rings were subjected to U-46619 (0.1 nM, 60 min)—groups c and d. Results (means ± SEM, *n* = 6) are expressed as a percentage of inhibition of the contraction induced by noradrenaline (0.1 μM), *p* < 0.05, two-way ANOVA with Tukey’s multiple comparisons test. Abbreviations: ACh, acetylcholine; SNP, sodium nitroprusside; CORM-2, carbon monoxide releasing molecule.

The vasodilator response to ACh; the exogenous NO donor SNP; the CO releasing molecule CORM-2; and the K_ATP_ channel opener pinacidil are presented in Figure 2A–D. Incubation with U-46619 (0.1 nM, 60 min) attenuated the vascular response to ACh, SNP, CORM-2, and pinacidil, see Table 1. *Myo*-inositol (1 µM, 60 min) protected arteries against the U-46619-induced effect, which resulted in a normalized response as observed in control arteries. On the other hand, arteries pre-incubated with *myo*-inositol under control conditions became more reactive to ACh and CORM-2 as compared to the control. This effect was not observed for SNP and pinacidil, see Table 1.

**Figure 3:**
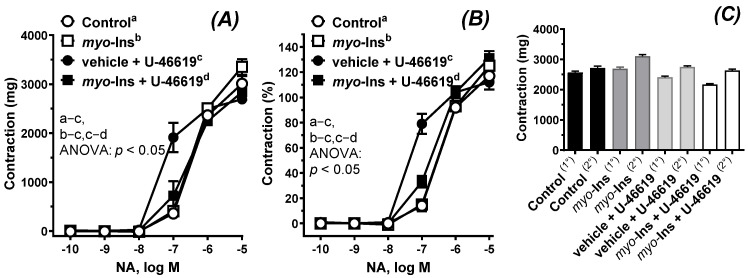
The cumulative concentration-response curves to noradrenaline (**A**,**B**) and 75 mM KCl (**C**) in the isolated thoracic arteries of the control (a), *myo*-inositol alone (b), vehicle plus U-46619 (c), *myo*-inositol plus U-46619 (d). Aortic rings were pre-incubated with either *myo*-inositol (1 µM, 60 min)—groups b and d or vehicle (KHS)—groups a and c. Next, aortic rings were subjected to U-46619 (0.1 nM, 60 min)—groups c and d. Contraction to KCl was performed before and after the 2-h pre-incubation period, (1°) or (2°), respectively. Results (means ± SEM, *n* = 6) are expressed as a percentage of the contraction induced by KCl (75 mM) and as mg of developed tension, *p* < 0.05, two-way ANOVA with Tukey’s multiple comparisons test.

Arteries subjected to U-46619 were more reactive to the vasoconstrictor NA (Figure 3A,B); and *myo*-inositol shifted that response towards control conditions, see Table 1. There was no significant difference in KCl-induced contraction between the analyzed groups (Figure 3C). 

Results are expressed as Emax (%) and logEC_50_, see Table 1.

### 3.3. Nitric Oxide Release

**Figure 4:**
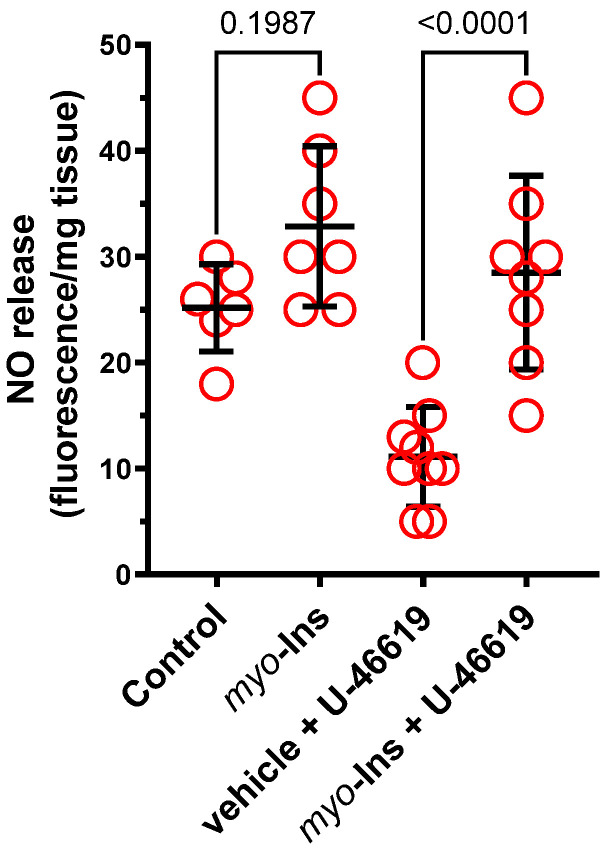
The effect of pre-incubation with *myo*-inositol (1.0 µM) on the basal release of NO in control arteries and arteries subjected to U-46619. Results (means ± SD) are expressed as arbitrary units of fluorescence (AU) per milligram of tissue. Number of animals: *n* = 6–8.

No significant difference in NO release was observed in the control (vehicle) and *myo*-inositol (1 µM) only groups (×1.3-fold, *p* = 0.1987). Incubation with U-46619 decreased (×0.4-fold, *p* = 0.0029) the NO production in the control group, which was not observed in *myo*-inositol treated arteries (×0.86-fold, *p* = 0.6053). *Myo*-inositol increased NO-production in U-46619 treated arteries (×2.6-fold, *p* < 0.0001); see Figure 4.

### 3.4. Detection of Hydrogen Peroxide

**Figure 5:**
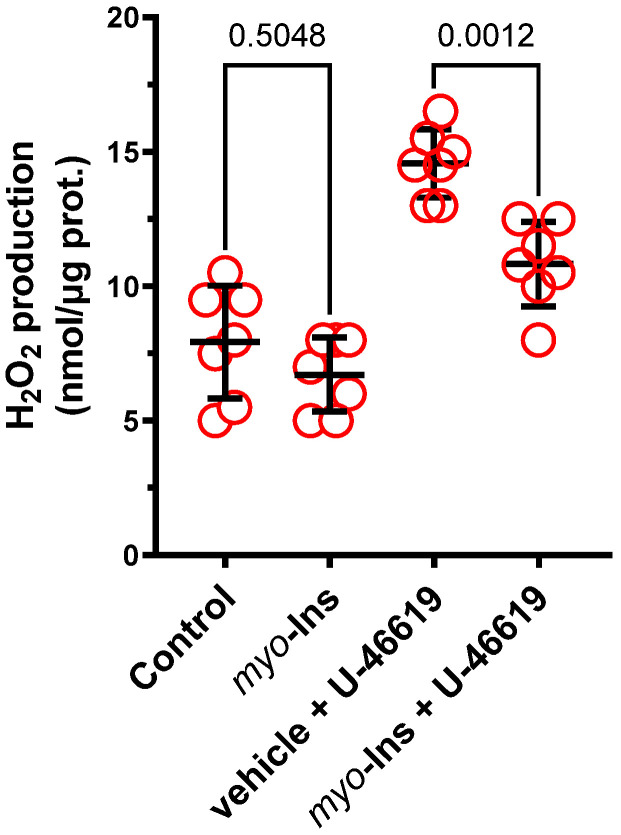
The effect of pre-incubation with *myo*-inositol (1.0 µM) on the production of hydrogen peroxide (H_2_O_2_) in control arteries and arteries subjected to U-46619. Results (means ± SD) are expressed as nanomoles of H_2_O_2_ *per* microgram of protein. Number of animals: *n* = 6–8.

No significant difference in the H_2_O_2_ content was observed in the control (vehicle) and *myo*-inositol (1 µM) only groups (×0.8, *p* = 0.5048). Incubation with U-46619 increased the H_2_O_2_ production under control conditions (×1.8-fold, *p* < 0.0001) and in *myo*-inositol incubated arteries (×1.6-fold, *p* = 0.0004). *Myo*-inositol decreased H_2_O_2_ production in U-46619 treated arteries (×0.7-fold, *p* = 0.0012), see Figure 5.

### 3.5. Markers of Oxidative Stress in Plasma

**Figure 6:**
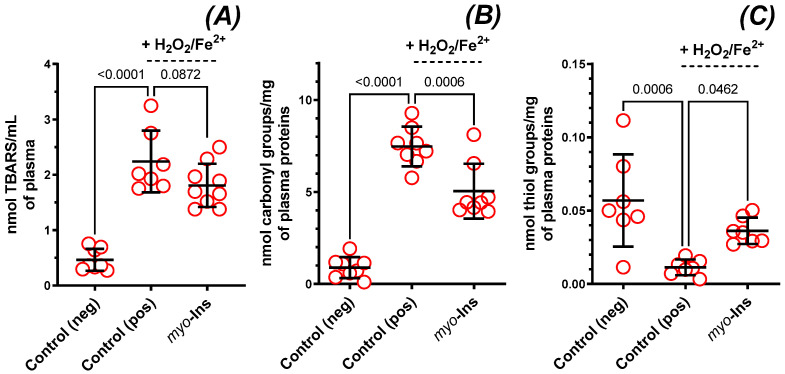
The effect of *myo*-inositol (1.0 µM, pre-incubation time—5 min, 37 °C) on the lipid peroxidation (**A**), and the oxidative damages of blood plasma protein measured as protein carbonylation (**B**), and the level of thiol groups (**C**) treated with H_2_O_2_/Fe^2+^ (incubation time—25 min, 37 °C). Results are given as means ± SD (*n* = 9). Control negative refers to the plasma not treated with H_2_O_2_/Fe^2+^, whereas control positive refers to the plasma treated with H_2_O_2_/Fe^2+^. One-way ANOVA followed by a Dunnett’s multiple comparisons test.

When human plasma was exposed to a strong pro-oxidant chemical agent (H_2_O_2_/Fe^2+^), it resulted in an enhanced plasma lipid peroxidation (TBARS, ×4.8-fold, *p* < 0.0001, Figure 6A), increased plasma protein carbonylation (×8.4-fold, *p* < 0.0001, Figure 6B), and a decrease in thiol groups (×0.2-fold, *p* = 0.0006, Figure 6C). *Myo*-inositol (1 µM) reduced the H_2_O_2_/Fe^2+^-induced plasma lipid peroxidation (result not significant: TBARS, ×0.8-fold, *p* = 0.0872, Figure 6A), reduced protein carbonylation (×0.7-fold, *p* = 0.0006, Figure 6B), and provided protection of the thiol groups (×3.2-fold, *p* = 0.046, Figure 6C).

### 3.6. Parameters of Hemostasis

**Figure 7:**
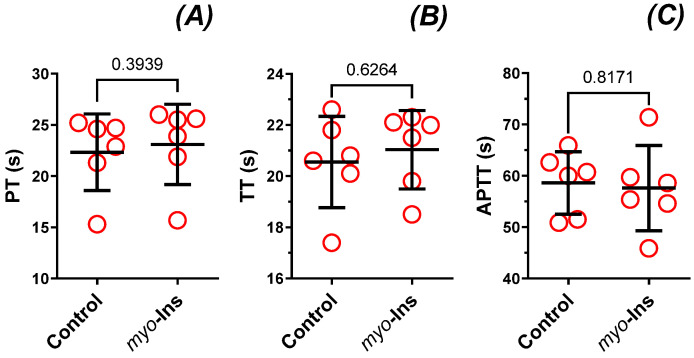
The effect of *myo*-inositol (1.0 µM, incubation time—30 min, 37 °C) on the hemostatic parameters: the prothrombin time (**A**), the thrombin time (**B**), and the activated partial thromboplastin time (**C**) of human plasma. Data are expressed as means ± SD (*n* = 6). Unpaired t test for TT and APTT, and Mann-Whitney test for PT. Abbreviations: APTT, activated partial thromboplastin time; PT, prothrombin time; TT, thrombin time.

No significant difference in either PT (×1.0-fold), TT (×1.0-fold), or APTT (×1.0-fold) was observed in the control and *myo*-inositol (1 µM) only groups (*p* ≥ 0.3939, Figure 7A–C).

## 4. Discussion

Overall, the presented results show that pre-incubation with *myo*-inositol (1 µM, 60 min) improves vascular response in compromised arteries subjected to a TxA_2_ receptor agonist (U-46619, 0.1 nM, 60 min). In addition, the increase in the NO release and/or bioavailability and decreased H_2_O_2_ production, and attenuated H_2_O_2_/Fe^2+^-induced oxidative damage of blood plasma protein, seem to be the mechanisms involved in the *myo*-inositol-induced effect under oxidative stress.

Our study demonstrated that *myo*-inositol did not accumulate into the structure of the thoracic arteries, as no significant difference within the studied groups (1, 10, 100 µM) and the control was observed.

Based on previous experimental protocols, ACh-induced vasodilation was studied in arteries with an intact endothelium, protected in a first step with *myo*-inositol (1 µM, 60 min) and then subjected to U-46619 (0.1 nM, 60 min), a synthetic PGH_2_ analog and TxA_2_ receptor agonist [23,24,25]. TxA_2_ is a potent vasoconstrictor which becomes activated during tissue injury and inflammation [16]. This pathological state disturbs the redox processes in cells which leads to increased ROS generation and decreased NO [24]. Exposure of these ROS to membrane proteins and lipids induces membrane peroxidation reactions and further generates more products which also activate the TxA_2_ receptors. TxA_2_ is also involved in the progression of myocardial infarction, atherosclerosis, coronary vascular spasticity, cardiovascular disease, asthma, kidney and liver disease, allergy, cancer, and inflammation [23,24].

In the aortic rings subjected to U-46619, the vascular relaxation to ACh, SNP, CORM-2 and pinacidil was reduced, which is a typical response observed in impaired arteries, and was also observed in arteries from either hypertensive or orchidectomized rats [24]. Our results show that *myo*-inositol improved vascular relaxation under control and U-46619-induced conditions. However, in impaired arteries due to U-46619 pre-incubation, the effect of *myo*-inositol was more potent, compared to the arteries without U-46619. This corresponded well with NO release, as we observed an increased release of NO in arteries pre-incubated with *myo*-inositol. The next step was to analyze the sensitivity of the smooth muscles to the exogenous NO, which is an endothelium independent mechanism. Our results show that the SNP-induced response was not altered with *myo*-inositol under the control conditions. However, the SNP-induced relaxation was impaired (the response was shifted to the right) under U-46619-stimulated conditions, and *myo*-inositol protected arteries against that change induced by U-46619. Thus, we have concluded that the endothelium-dependent cyclic guanosine monophosphate (cGMP) pathway was not modified in a significant way under *myo*-inositol pre-treatment under control conditions, as it was by the U-46619, which has also been reported in rat thoracic arteries [24] and radial arteries [26]. Interestingly, *myo*-inositol improved the above-mentioned response in U-46619 treated arteries, which was dependent on the vessel’s conditions. The results described here indicate that *myo*-inositol may act through endothelium dependent (U-46619 stimulation) and independent (control conditions) mechanisms.

Since the mechanism of action of CO is like that of NO, we have analyzed the vasodilation to this gas mediator. The results showed that the vasodilator response induced by the CO releasing molecule, CORM-2, was potentiated by *myo*-inositol. Like the above-mentioned effect of *myo*-inositol in an ACh-induced response, CORM-2 also induced a less pronounced increase in vasodilation in the aortic rings without U-46619 compared to the arteries subjected to U-46619. Since NO and CO share a similar mechanism of action through the hyperpolarization of the cell membranes [24], we studied the influence of K_ATP_ channels in vasodilation induced by *myo*-inositol. Our results showed that *myo*-inositol improved the response to the K_ATP_ channel opener in arteries subjected to U-46619, which was not observed under control conditions. This points to the decreased participation of a hyperpolarizing mechanism by the activation of TxA_2_ receptors, which has also been described for rat thoracic and pulmonary arteries [24]. Further studies are needed to describe how *myo*-inositol achieves this specific effect.

We also analyzed the effect of *myo*-inositol on the contraction induced by two different mechanisms, the ones induced by KCl and NA. High KCl induces membrane depolarization and calcium entry through L-type voltage-operated calcium channels (L-VOCCs) in the smooth muscles, meanwhile NA is the agonist of alpha adrenoreceptors using two second messengers: IP_3_ (inositol trisphosphate) and DAG (diacylglycerol). IP_3_ diffuses and stimulates calcium release from the endoplasmic reticulum, meanwhile DAG activates protein kinase C. Our results have demonstrated, for the first time, that the response to KCl and NA were not modified under the control conditions with *myo*-inositol pre-incubation. However, NA-induced contraction was shifted to the left after U-46619-incubation, and *myo*-inositol shifted that response back to the right. In the presented experiment, the incubation period was 2-h, and it is possible that *myo*-inositol, by reducing different ROS, improves contraction in U-46619 treated arteries through the modulation of calcium influx and myofilament function, which are impaired by aging [27]. Therefore, the next step was to analyze the content of H_2_O_2_ in rat thoracic arteries, and the results showed that pre-incubation with *myo*-inositol decreased the production of this particular ROS.

Hemostatic parameters and oxidative stress markers are commonly studied to determine the efficacy of new anti-thrombotic medications or supplements. The testing of hemostatic parameters may help to diagnose the reason for blood clot-formation and explain the cause of excessive bleeding [28]. In this work, we studied for the first time the influence of *myo*-inositol on H_2_O_2_/Fe^2+^-induced oxidative stress in human blood plasma in vitro. *Myo*-inositol at the applied concentration (1 µM) did not protect against lipid peroxidation, however, it provided protection against protein degradation. *Myo*-inositol significantly reduced the carbonylation of plasma proteins and provided protection of the thiol groups. A similar decrease in ROS generation was reported in an in vitro model of human umbilical vein endothelial cells (HUVEC), however with a x1000-fold higher concentration of *myo*-inositol [4]. Moreover, we examined the selected hemostatic parameters of blood plasma and found that *myo*-inositol neither modified the activated partial thromboplastin time (APTT), the prothrombin time (PT), nor the thrombin time (TT). 

## 5. Conclusions

This is the first detailed study of the biological activity of a low *myo*-inositol dose (1 µM) under increased oxidative stress in vitro. We observed that the biological properties of *myo*-inositol improved vascular response under shared stress due to U-46619 exposure and influenced NO release and H_2_O_2_ production in the vasculature. The activation of ATP dependent potassium channels was involved in the observed vascular relaxation. Moreover, *myo*-inositol at the applied concentration protected blood plasma proteins, but not lipids, against peroxidation processes in vitro, even though it had not accumulated in the aortic structure. Thus, *myo*-inositol might be recommended as a functional food product, however, the real curative effects must be verified with the use of in vivo studies. 

## Figures and Tables

**Table 1:** Vascular response to the vasodilators: acetylcholine (ACh), sodium nitroprusside (SNP), CO releasing molecule (CORM-2), pinacidil, and the vasoconstrictor noradrenaline (NA).

Group	Control (Vehicle)		*Myo*-Inositol (1 µM)		Vehicle + U-46619 (0.1 nM)		*Myo*-Inositol + U-46619	
	Emax (%)	LogEC_50_	Emax (%)	LogEC_50_	Emax (%)	LogEC_50_	Emax (%)	LogEC_50_
ACh	76.36 ^ac^	−6.785 ^a^	91.40 ^a^	−7.347 ^a^	58.47 ^bc^	−6.498 ^b^	86.69 ^b^	−6.834 ^b^
±SEM	2.023	0.070	2.384	0.056	0.807	0.032	1.879	0.058
SNP	103.7	−8.186 ^c^	106.9	−8.363	101.1	−7.478 ^bc^	104.3	−8.076 ^b^
±SEM	3.835	0.114	3.4	0.108	4.861	0.137	1.646	0.088
CORM-2	83.08 ^a^	−4.733	95.61 ^a^	−4.878	44.08 ^bc^	−4.676	83.29 ^b^	−4.779
±SEM	0.040	0.138	2.800	0.030	0.014	0.078	4.240	0.057
Pinacidil	97.72	−6.977 ^c^	100.7	−6.906	92.49	−6.489 ^bc^	97.67	−6.988 ^b^
±SEM	3.162	0.106	4.876	0.149	6.711	0.165	3.608	0.130
NA	125.1	−6.359 ^c^	133.2	−6.314	112.9 ^b^	−7.264 ^bc^	135.1 ^b^	−6.525 ^b^
±SEM	6.744	0.113	5.493	0.084	7.268	0.171	2.074	0.035

^a^ Control (vehicle) vs. *myo*-inositol, ^b^ vehicle + U-46619 vs. *myo*-inositol + U-46619, ^c^ Control vs. U-46619, the same superscript letters within the rows differ in a significant way, *p* < 0.05.

## Data Availability

Data is available from the corresponding author upon the request.
